# Effect of bilevel continuous positive airway pressure for patients with type II respiratory failure due to acute exacerbation of COPD: A protocol for systematic review and meta-analysis

**DOI:** 10.1097/MD.0000000000024016

**Published:** 2021-01-15

**Authors:** Wenzhe Teng, Hu Chen, Siyao Shi, Yin Wang, Kangyao Cheng

**Affiliations:** a Shanghai University of Traditional Chinese Medicine; b School of Nursing, Shanghai University of Traditional Chinese Medicine, Shanghai, China.

**Keywords:** bilevel continuous positive airway pressure, chronic obstructive, pulmonary disease, respiratory insufficiency, systematic review

## Abstract

**Background:**

The purpose of this study is to determine the therapeutic efficacy of bi-level continuous positive airway pressure (BIPAP) intervention in patients with type II respiratory failure due to acute exacerbation of chronic obstructive pulmonary disease (COPD).

**Methods:**

This review will only include randomized controlled trials (RCTs). The search strategy will be applied to 4 Chinese databases: China National Knowledge Infrastructure (CNKI), Wanfang Database, Chinese Science and Technology Journal Database (VIP), and Chinese Biomedical Literature Database (SinoMed); and 5 foreign literature databases: PubMed, Cochrane Library, Springer, EBSCO, and Web of Science. RCTs published from inception to October 2020 will be included. The 2 researchers will independently screen and extract the data and assess quality. The main results obtained through blood gas analysis and equipment observation, heterogeneity assessment, sensitivity analysis, funnel chart synthesis, data synthesis, and grouping analysis will be carried out using Review Manager 5.4 software. The trial sequential analysis will be completed using TSA v0.9 developed by the CTU at the Copenhagen Clinical Trial Center.

**Results:**

In the current meta-analysis, we will provide more practical and targeted results for the therapeutic efficacy of BIPAP in patients with type II respiratory failure due to acute exacerbation of COPD.

**Conclusion:**

This study will provide new evidence for the therapeutic efficacy of BIPAP in patients with type II respiratory failure due to acute exacerbation of COPD.

**Registration number:**

INPLASY2020110003 (DOI:10.37766/inplasy2020.11.0003).

## 1 Introduction

### 1.1 Description of condition

The COVID-19 epidemic has again brought respiratory diseases to the forefront. As a representative of respiratory pathology, the incidence of chronic obstructive pulmonary disease (COPD) has been high. Data shows that there are currently more than 600 million people living with COPD in the world, COPD has become the fifth largest burden in the world,^[1]^ and global deaths due to COPD are increasing year-by-year. In 2012, more than 3 million people died of COPD worldwide. It has been predicted that COPD will become the 3rd leading cause of death worldwide by the year 2020.^[2–4]^ COPD is divided into the stable phase and acute exacerbation of COPD (AECOPD),^[2]^ and AECOPD is the critical period in the course of COPD. One study found that the in-hospital mortality rate for AECOPD patients was 11%, and the mortality rate 2 years after discharge was 49%.^[5]^ Due to the impairment of expiratory capacity in AECOPD, CO_2_ retention can easily lead to type II respiratory failure, which causes not only respiratory acidosis but also rapid loss of lung function. Type II respiratory failure due to AECOPD can greatly increase mortality^[6]^ and puts the patient at severe risk.

### 1.2 Description of intervention

Patients with type II respiratory failure due to AECOPD experience deterioration of lung function and retention of CO_2_; therefore, medication and respiratory support therapy are required. Respiratory support includes oxygen therapy, high-flow oxygen therapy, non-invasive ventilation (NIV), and invasive mechanical ventilation. NIV is the first choice for AECOPD patients without related contraindications because of benefits such as improving gas exchange and reducing the loss of respiratory function.^[2]^ NIV mainly includes 2 modes: continuous positive airway pressure (CPAP) and noninvasive pressure support ventilation (NIPSV). Bi-level continuous positive airway pressure (BIPAP) is based on noninvasive pressure support ventilation in NIV. The BIPAP gas volume varies dynamically in each respiratory cycle.^[7]^ The advantages of BIPAP in patients with type II respiratory failure due to AECOPD are avoidance of repeated inhalation of exhaled gas, reduction of CO_2_ retention, and correction of acid-base imbalance. BIPAP can fully rest the respiratory muscles to prevent further deterioration of lung function.

In the studies of Carrera,^[8]^ Castillo,^[9]^ and Khilnani,^[10]^ it was found that BIPAP could significantly improve respiratory acidosis and pulmonary function compared with conventional oxygen therapy; however, Barb et al^[11]^ found no significant difference between the 2 methods. At present, there is no corresponding recommendation for BIPAP in type II respiratory failure due to AECOPD in the relevant guidelines.^[2,12]^

### 1.3 Objective of this study

The purpose of this study is to explore the effect of BIPAP on pulmonary function and CO_2_ retention in patients with respiratory failure due to AECOPD, and to provide reliable evidence for related clinical practice.

## 2 Methods

The protocol was registered on the International Platform of Registered Systematic Review and Meta-analysis Protocols (INPLASY2020110003). The preferred reporting items for systematic review and meta-analysis protocols (PRISMA) will serve as guidelines for reporting present review protocol and subsequent formal paper.

### 2.1 Inclusion criteria for study selection

#### 2.1.1 Types of studies

We will only choose randomized controlled trials (RCTs); other study designs, including non-randomized controlled trials, will be excluded.

#### 2.1.2 Types of participants studied

All participants were adults (aged >18 years) meeting the following criteria:

1. diagnosis of COPD according to the COPD clinical guidelines;
2. experiencing AECOPD;
3. according to blood gas analysis, pH < 7.35 and partial pressure of arterial CO_2_ (PaCO_2_) >50 mm Hg;
4. unobstructed respiratory tract, adequate strength for spontaneous breathing, and absence of pneumothorax, pneumomediastinum, and lung bullae;
5. no facial deformity or facial triangle infection;
6. no severe cardiac, hepatic, or renal insufficiency, and no hemodynamic instability;
7. no malignant tumor or severe brain disease; and
8. conscious with no mental illness.

#### 2.1.3 Type of intervention

All participants will receive standard treatment, including bronchodilators, glucocorticoids, antibiotics, low flow oxygen therapy, and if necessary respiratory stimulants will be used to treat. The experimental group will receive BIPAP + routine treatment, generally selecting S/T mode. The inspiratory pressure (IPAP) will be greater than the expiratory pressure (EPAP), the oxygen flow rate will be adjusted to ensure an arterial oxygen saturation (SaO_2_) greater than 90%, and the daily duration of treatment will be more than 5 hours.

#### 2.1.4 Outcome measures

##### 2.1.4.1 Main outcome indicators

These include results of blood gas analysis, including partial pressure of arterial oxygen (PaO_2_), PaCO_2_, and pH; intubation rate; and pulmonary function, including forced expiratory volume in 1 second and forced expiratory volume in 1 second/forced vital capacity.^[13]^

##### 2.1.4.2 Secondary outcome indicators

These include heart rate, respiratory rate, hospitalization time, incidence of complications, blood pressure, and mortality.

#### 2.1.5 Search strategy

The search strategy for this study will be applied to 4 Chinese databases: China National Knowledge Infrastructure (CNKI), Wanfang Database, Chinese Science and Technology Journal Database (VIP), and Chinese Biomedical Literature database (SinoMed); and 5 foreign literature databases: PubMed, Cochrane Library, Springer, EBSCO, and Web of Science. All the English and Chinese literature published from inception to October 2020 will be included. The search strategy for PubMed is shown in Table 1, and searches in other databases also follow this search strategy.

**Table 1:** Search strategy for Pubmed.

Number	Search Detail
1	MeSH descriptor: [pulmonary disease, chronic obstructive] explode all trees
2	COPD OR COAD
3	Obstructive Pulmonary Disease OR Obstructive Airway Disease OR Obstructive Lung Disease OR Airflow Obstruction^∗^
4	Chronic
5	#3 AND #4
6	#1 OR #2 OR #5
7	MeSH descriptor: [respiratory insufficiency] explode all trees
8	Respiratory Failure OR Respiratory Depression OR Ventilatory Depression
9	#7 OR #8
10	MeSH descriptor: [continuous positive airway pressure] explode all trees
11	CPAP Ventilation OR Positive Airway Pressure OR Biphasic Continuous Positive Airway Pressure OR Bilevel Continuous Positive Airway Pressure OR Airway Pressure Release Ventilation OR nCPAP Ventilation OR Ventilation Mode, APRV
12	#10 OR #11
12	#6 AND #9 AND #12

### 2.2 Data collection and extraction

#### 2.2.1 Selection of studies

**Figure 1:**
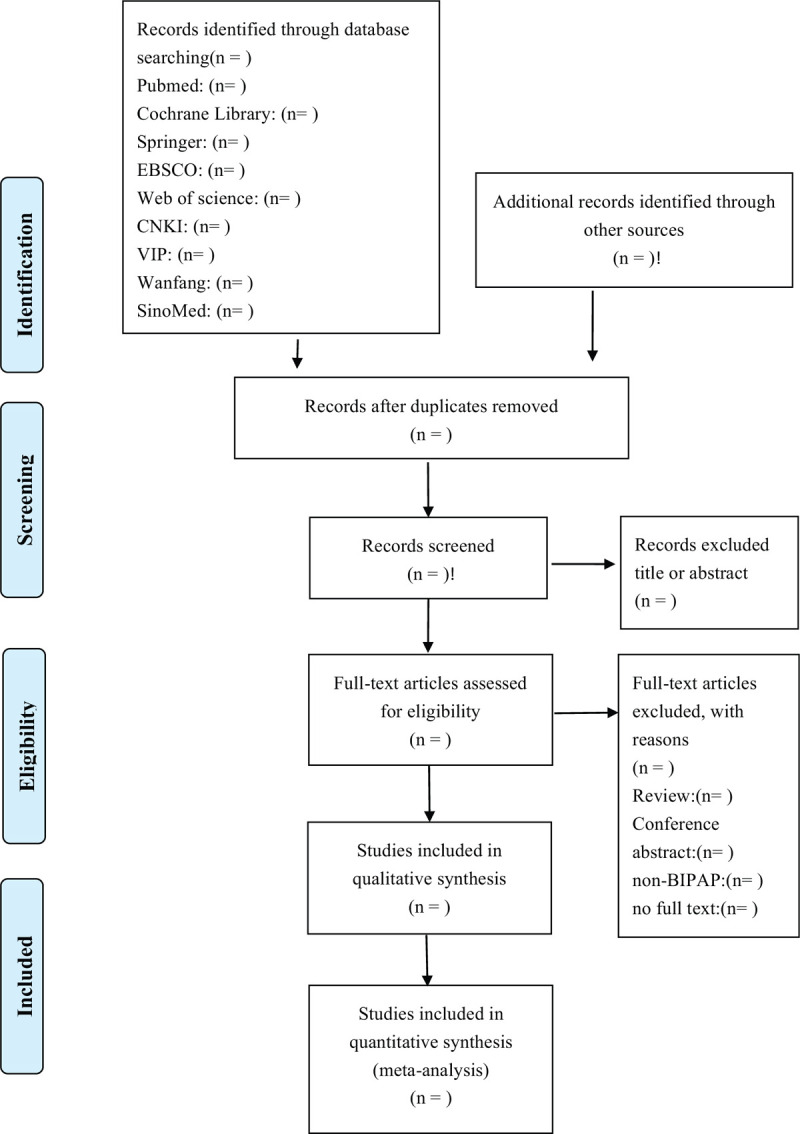
The PRISMA flow diagram shows the details of the research selection. It is divided into 4 parts (Identification, Screening, Eligibility, Included), and the steps of research screening are shown through the arrows. CNKI = Chinese National Knowledge Infrastructure, n = the number of studies, SinoMed = Chinese Biomedical Literature Database, VIP = Chinese Science and Technology Periodical Database.

The literature will be searched independently by 2 researchers who have participated in a series of evidence-based courses, selecting titles and abstracts according to the inclusion criteria and excluding irrelevant studies. If it is necessary to read the full text to decide whether a study is included, reviews, conference summaries, and studies with incomplete data must be excluded in the process, and if there is a dispute, the decision will be made by a third researcher who has achieved the training qualifications of the Australian JBI Evidence-based Health Care Centre. For questionable data, we will contact the author through e-mail for consultation. The details of research selection are shown in the PRISMA flow diagram (Fig. 1).^[14]^

#### 2.2.2 Data and information extraction

Data will be extracted and duplicated by 2 independent researchers. Detailed data and information will be extracted in the following forms: basic information (first author, year of publication, country), type of study, main characteristics of participants (age, course of disease, physiological indicators, sample size), intervention measures (intervention mode, duration of intervention), and main outcome.

#### 2.2.3 Addressing missing data

If the data are incomplete or missing, we will contact the author by email to gain the information. In the case of unavailable data, we will exclude the study.

#### 2.2.4 Evaluation of research quality

Two researchers who attended and graduated from a series of evidence-based courses will independently use the Australian Joanna Briggs Institute Center critical appraisal tool for RCTs^[15]^ to evaluate each of the 13 quality areas included in the literature. These include random grouping, allocation concealment, baseline comparability, subject blind method, intervention blind method, evaluator blind method, full follow-up, comparison of other intervention measures, reliability of evaluation methods, comprehensive result analysis, same evaluation methods, credibility of data analysis methods, and reasonableness of research and design. The quality level of each study will be assigned a grade of A, B, C, or D from high quality to low. Any divergence will be resolved through discussion and negotiation with the third researcher.

### 2.3 Statistical analysis

#### 2.3.1 Assessment of report bias

Funnel charts will be used to assess the potential for study bias, and the results of the assessment will be explained using Review Manager 5.4 charting.

#### 2.3.2 Assessment of heterogeneity

The Q test will be used to qualitatively determine inter-study heterogeneity; if *P* ≥ .1, there is no inter-study heterogeneity. For the included study, *I*^2^ statistics will be used to quantify the statistical heterogeneity and to evaluate the heterogeneity of the study.

#### 2.3.3 Measurement of treatment effect

We will use Review Manager 5.4 software for data analysis. For continuous variables, we will calculate the mean difference (MD) when the parameters of ventilator and blood gas analysis equipment are different in the study; we may calculate the standard mean difference with 95% confidence interval (CI). For dichotomous variables, such as intubation rate, the risk ratio with 95% confidence interval will be calculated. When the Q-test result shows *P* < .1, it indicates inter-study heterogeneity. For the included study, *I*^2^ statistics will be used to quantify the statistical heterogeneity. When *I*^2^ < 50%, the heterogeneity is considered acceptable, and the fixed effect model will be adopted. When *I*^2^ > 50%, the heterogeneity is considered significant, and sensitivity analysis and subgroup analysis to explore the source of heterogeneity is needed. If there is no significant clinical heterogeneity, a random effect model will be used for analysis; otherwise, descriptive analysis will be conducted.

#### 2.3.4 Subgroup analysis

If there is significant heterogeneity between the results, we will conduct a subgroup analysis of BIPAP inspiratory pressure (IPAP), time point of the measured results after intervention (<1 day vs >1 day), duration of intervention (<15 hour/day vs >15 hour/day), and age of the participants (<60 vs >60 years).^[2]^

#### 2.3.5 Sensitivity analysis

We will rule out the combined study one-by-one for sensitivity analysis to observe whether there is a significant change in the comprehensive results. If so, the removed study may affect the overall synthesis results, and we will re-evaluate the results carefully for merging.

#### 2.3.6 Trial sequential analysis

Meta-analysis is an accumulation of multiple trial results, but it can increase random errors and exaggerate the efficacy of intervention. Trial sequential analysis is used for related verification.^[16]^ This study will use TSA v0.9, developed by the CTU of the Copenhagen Clinical Trial Center, to complete the analysis. Comparing the sample size with the amount of information is required to determine whether the sample size is illustrative. The influence of random error is explained by judging the boundary value of trial sequential analysis formed by correction and the significant horizontal line and the cumulative Z-value curve of the meta-analysis.

#### 2.3.7 Quality of evidence

We will use GRADE (Grading of Recommendations, Assessment, Development, and Evaluation) to evaluate the quality of this study.^[17]^ According to GRADE, we will divide the results into 4 levels: high, medium, low, and very low, so as to evaluate the evidence quality of the study and reflect whether the study can provide reliable recommendations.

### 2.4 Ethical review and dissemination

Systematic reviews do not require ethical approval because individual data are not used. The results of this study will provide a reliable basis for the application of BIPAP in patients with type 2 respiratory failure due to AECOPD, and are also of great significance to clinical practice and research.

## 3 Discussion

The literature on the intervention effect of BIPAP in patients with type II respiratory failure due to AECOPD is controversial. Studies have provided evidence that early use of BIPAP can effectively alleviate deterioration and speed recovery of patients with type II respiratory failure due to AECOPD.^[8–10]^ However, some studies have shown that BIPAP is less effective than conventional therapy in these patients.^[11]^ This meta-analysis combines available evidence based on the widespread use of BIPAP in these patients to measure the degree of remission attributable to intervention with BIPAP. It will determine whether the existing BIPAP non-invasive ventilation techniques are more effective and reliable in patients with type II respiratory failure due to AECOPD.

This meta-analysis attempts to compare the differences in physiological indexes among participants at different time points after BIPAP intervention. For example, the studies of the Collaborative Research Group^[18]^ and Carrera^[8]^ measured outcome indexes such as blood gas values <24 hours and ≥24 hours after BIPAP intervention. In addition, the meta-analysis included some RCTs with innovative techniques, such as the newly designed sham BIPAP ventilator in the Carrera study,^[8]^ which was provided to the control group for intervention. The physical appearance of the sham BIPAP ventilator is the same as that of BIPAP, but it can only provide oxygen therapy (which has been verified by experiments). This design is different from the conventional intervention in the control group. It can make double blinding more achievable in most studies.

Studies have shown that not all modes of NIV are suitable for respiratory failure due to AECOPD.^[19–21]^ BIPAP is based on the NIPSV mode in NIV. The gas volume of BIPAP is dynamic,^[7]^ and its inspiratory positive airway pressure (IPAP) can overcome airway resistance and increase alveolar ventilation. Expiratory positive airway pressure (EPAP) promotes CO_2_ exhalation for patients with respiratory failure due to AECOPD.^[22]^ However, there are no official recommendations in this regard; therefore, this study will attempt to explore the effect of BIPAP on pulmonary function and CO_2_ retention in patients with type II respiratory failure due to AECOPD, so as to provide a reliable basis for clinical application.

## Author contributions

**Conceptualization:** Wenzhe Teng, Hu Chen, Kangyao Cheng.

**Data curation:** Wenzhe Teng, Hu Chen, Siyao Shi.

**Formal analysis:** Wenzhe Teng.

**Funding acquisition:** Kangyao Cheng.

**Investigation:** Hu Chen, Siyao Shi.

**Literature retrieval:** Wenzhe Teng, Hu Chen and Siyao Shi.

**Methodology:** Kangyao Cheng.

**Resources:** Wenzhe Teng.

**Supervision:** Yin Wang, Kangyao Cheng.

**Validation:** Wenzhe Teng.

**Visualization:** Wenzhe Teng.

**Writing – original draft:** Wenzhe Teng.

**Writing – review & editing:** Wenzhe Teng.

## Abbreviations

Abbreviations: AECOPD = Acute exacerbation of COPD, BIPAP = Bilevel Continuous Positive Airway Pressure, COPD = Chronic Obstructive Pulmonary Disease, EPAP = expiratory positive airway pressure, GRADE = Grading of Recommendations, Assessment, Development, and Evaluation, IPAP = inspiratory positive airway pressure, NIV = Non-invasive ventilation, RCT = randomized controlled trial.
